# CD31 and VEGF are prognostic biomarkers in early-stage, but not in late-stage, laryngeal squamous cell carcinoma

**DOI:** 10.1186/s12885-018-4180-5

**Published:** 2018-03-09

**Authors:** Anke Schlüter, Patrick Weller, Oliver Kanaan, Ivonne Nel, Lukas Heusgen, Benedikt Höing, Pia Haßkamp, Sebastian Zander, Magis Mandapathil, Nina Dominas, Judith Arnolds, Boris A. Stuck, Stephan Lang, Agnes Bankfalvi, Sven Brandau

**Affiliations:** 1 Department of Otorhinolaryngology, Head and Neck Surgery, University Hospital Essen, Essen, Germany; 2 Molecular Oncology Risk-Profile Evaluation, Department of Medical Oncology, West German Cancer Center, University Duisburg-Essen, 45122 Essen, Germany; 3 Institute for Pathology, University Hospital Essen, Essen, Germany; 4 Present address: ABA GmbH & Co.KG, BMZ2, 44227 Dortmund, Germany; 5 Present address: Martha-Maria Hospital Munich Solln, Munich, Germany; 6 Present address: Department of Otorhinolaryngology, Head and Neck Surgery, Asklepios Kliniken Hamburg, Hamburg, Germany; 7 Present address: Department of Otorhinolaryngology, Head and Neck Surgery, University Hospital Marburg, Marburg, Germany; 8 Experimental and Translational Research, Department of Otorhinolaryngology, University Hospital Essen, Hufelandstrasse 55, 45147 Essen, Germany

**Keywords:** Angiogenesis, Laryngeal squamous cell carcinoma, Biomarkers, VEGF, CD31

## Abstract

**Background:**

Patients suffering from squamous cell carcinoma of the larynx (LSCC) with lymphatic metastasis have a relatively poor prognosis and often require radical therapeutic management. The mechanisms which drive metastasis to the lymph nodes are largely unknown but may be promoted by a pro-angiogenic tumor microenvironment. In this study, we examined whether the number of microvessels and the expression level of vascular endothelial growth factor (VEGF) in the primary tumor are correlated with the degree of lymph node metastasis (N-stage), tumor staging (T) and survival time in LSCC patients.

**Methods:**

Tissue-Microarrays of 97 LSCC patients were analyzed using immunohistochemistry. The expression of VEGF was scored as intensity of staining (low vs high) and the number of CD31-positive vessels (median </≥7 vessels per visual field) was counted manually. Scores were correlated with N-stage, T-stage and 5-year overall survival rate.

**Results:**

A high expression of angiogenic biomarkers was not associated with poor overall survival in the overall cohort of patients. Instead high CD31 count was associated with early stage cancer (*p* = 0.004) and in this subgroup high VEGF expression correlated with poor survival (*p* = 0.032). Additionally, in early stage cancer a high vessel count was associated with an increased recurrence rate (*p* = 0.004).

**Conclusion:**

Only in the early stage subgroup a high expression of angiogenic biomarkers was associated with reduced survival and an increased rate of recurrence. Thus, biomarkers of angiogenesis may be useful to identify high risk patients specifically in early stage LSCC.

## Background

Head and neck carcinomas with lymphatic metastasis usually require radical therapeutic management and are associated with poor prognosis. This is particularly true for squamous cell carcinoma of the larynx (LSCC). In addition, patients with lymph node (LN) involvement have a worse prognosis compared to patients without LN metastasis [1]. The identification of early stage LSCC patients at high risk therefore could help improving prognosis and treatment selection and preventing recurrence [2, 3]. Although clinically of high importance, the mechanisms which drive metastasis to the lymph nodes in LSCC are still largely unknown. It has been suggested that a pro-angiogenic tumor microenvironment may promote lymphatic metastasis [4–6]. The presence of neovascularization around neoplastic tissue is a typical finding in many solid tumors. Angiogenesis seems to be an important biological parameter implicated in tumor growth, metastasis and progression [4, 5]. However, in LSCC controversial data with respect to the impact of angiogenesis on metastasis have been reported. Some studies suggested angiogenesis as a precursor for regional lymph node metastasis [6] and implicated microvessel density in local tumor progression [7]. In contrast, other studies did not show any prognostic relation between angiogenesis and prognosis, especially in patients with LSCC [8–11].

Well defined markers of angiogenesis are CD31 and vascular endothelial growth factor (VEGF). CD31 is highly expressed on the surface of endothelial cells and well established for the monitoring of vessel density in malignant tissue. It is a member of the Immunoglobulin-superfamily PECAM-1 [12] and it was reported that CD31 is involved in angiogenesis for example in early breast cancer [13]. CD31 was even used as a prognostic marker for nasopharyngeal cancer [14]. Sion-Vaardy et al. found a significantly increased number of vessels in head and neck tumors with deeper invasion [15]. Kyzas et al., however, have questioned the role of CD31 for the prognosis of LSCC in their study on 69 patients with LSCC and oral cancer. They rather suggested that the expression of VEGF might have prognostic significance in these patients [16].

VEGF is an important growth factor and signaling molecule involved in vasculogenesis and angiogenesis [17]. VEGF was reported to influence the pathogenesis of LSCC as a parameter of angiogenesis [16, 18–21]. In addition, a gene polymorphism of VEGF was suspected to be a risk factor for LSCC [22]. Chen et al. found as well that VEGF influenced the pathogenesis of head and neck cancer and supposed an important role of VEGF as a serological biomarker [23]. In contrast, Burian et al. were not able to demonstrate a relevant impact of elevated VEGF in the prognostic relevance of LSCC [8].

The controversial results regarding the role of these angiogenic factors may be due to the heterogeneity of the patient cohorts with regard to early or late stage disease. The aim of this study therefore was to investigate a possible relation between markers of angiogenesis, N-stage, T-stage, overall survival and recurrence rates in a cohort of 97 patients with LSCC with a special emphasis on tumor stage.

## Methods

### Study population and ethics approval

Patients with LSCC treated in the Department of Otorhinolaryngology, Head and Neck Surgery at the University Hospital Essen between 1995 and 2004 were included in this study. Samples of tumor tissue were collected during diagnostic biopsies or tumor resection and were stored for later analysis. Preparation of tissue and immunohistochemistry were performed between March 2014 and December 2014. Patient characteristics and data on survival were retrospectively assessed by extracting the corresponding data from the patient medical charts. Analysis of experimental data was performed in a completely anonymous fashion. The study was approved by the local ethics committee of the Medical Faculty of the University Duisburg-Essen (12–5192-BO) and was performed according to Declaration of Helsinki. Based on the retrospective and anonymous character of the study the approval contained a waiver for written informed consent. Tumor staging was performed according to the criteria of the Tumor-Node-Metastasis staging system, first reported by Pierre Denoix in the 1940’s and adapted and compiled by the International Union against Cancer (UICC) [24]. Treatment regimens were planned according to tumor stage, surgical possibilities and patients´ decision. Patients with low T stage (T1 and T2) underwent mostly surgical therapy (70%). For patients with high T-stage (T3 + T4) surgery followed by RTX was chosen in the majority of cases (66%); more details on the treatment regimens are listed in Table 1.

**Table 1:** Patient Demographics

	Patients (*n* = 97)	
Demographic	No.	%
Tumor Staging		
T1	21	21
T2	29	30
T3	24	25
T4	23	24
Lymph nodes (N-stage)		
N0	67	70
N1	9	9
N2	20	20
N3	1	1
Gender		
male	86	89
female	11	11
Therapeutical Treatment		
Surgery	44	45
Surgery+RCTX	8	8
Surgery+RTX	40	41
RCTX	2	2
RTX	3	3
	Patients (*n* = 59)	
Recurrence No. of Patients	yes	no
T1	3	14
T2	9	9
T3	3	7
T4	1	13
Distribution Data (Total no.)	VEGF	CD31
No. of patients (97)	87	89
Recurrence total (59)	50	52
T-stage low (50)	42	46
Recurrence T-stage low (34)	27	30
N0 (67)	58	59
Recurrence N0 (44)	39	41

Patients with LSCC who were treated between 1995 and 2004 in our department were consecutively included in this study (*n* = 97). Distribution of T-stage, N-stage, gender and treatment of the patients is shown. For patients with available recurrence data a correlation between expression of VEGF/CD31 and the rate of recurrence during 5 year follow up was performed. The lower panel of the table indicates the number of patients eligible for analysis of recurrence in the respective groups. Recurrence was defined as local relapse or cervical lymph node metastasis within 5 years after diagnosis. Number of recurrences and break-up according to T stage are also shown

Recurrence was defined as local relapse or cervical lymph node metastasis within 5 years after diagnosis. Overall recurrence rate was 27% (16 out of 59 patients from whom recurrence data were available, Table 1).

### Preparation of tissue microarrays and immunohistochemistry

**Fig. 1:**
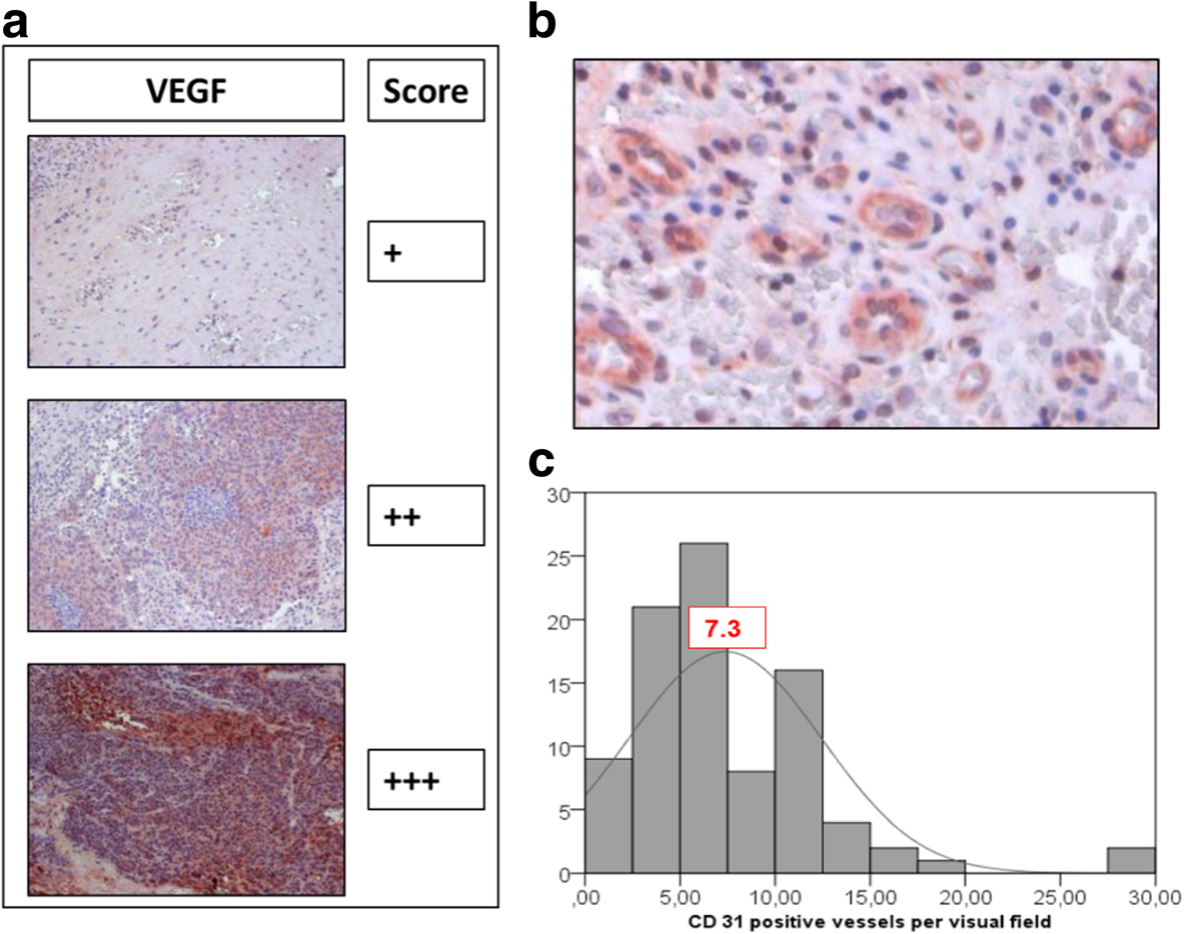
Immunohistochemical staining and analysis. Immunohistochemical staining of VEGF (**a**) and CD31 (**b**) was evaluated. The immune reactive score (IRS) of VEGF was calculated as intensity of the staining reaction, evaluated by three independent observers. VEGF was scored as absent (−), *weak (+)*, *medium (++)*, and *strong (+++)* (Magnification = 200×) and categorized in low and high. Low includes −/+ and high includes ++/+++. CD31 was scored by absolute vessel counting per visual field (**b**). The median of the counted CD31-positive vessels was 7.3 per visual field (**c**) and was used as a cut-off to categorize patients into low (< 7) vs high (≥7)

For preparation of tissue microarrays whole slides were inspected by a trained pathologist and regions of interest containing malignant tissue areas were marked. The tissue cores of 3 mm thickness were extracted from formalin-fixed/paraffin-embedded tumor tissue blocks using a skin biopsy punch (PFM, Cologne, Germany) and cut into 5 μm sections. Subsequently, the cores were de-parafinised and the antigens were retrieved by HIAR (heat-induced antigen retrieval) in citrate buffer pH 6.0 (Sigma–Aldrich, Taufkirchen, Germany). Samples were incubated with the primary antibodies (monoclonal mouse anti-human CD31, eBioscience, San Diego, USA and polyclonal rabbit anti-human VEGF, Millipore, Schwalbach, Germany) at 4 °C overnight. Then samples were incubated with peroxidase-coupled secondary antibodies for 30 min at room temperature and developed with AEC solution (Invitrogen, Karlsruhe, Germany) for 10 min. Nuclei were stained with haematoxylin (Carl Roth, Karlsruhe, Germany) for 1 min. Samples were covered with Kaisers glycerin gelatin (Merck, Darmstadt, Germany) and analyzed with a Zeiss Axioscope 2 microscope (Zeiss, Jena, Germany) at 200-fold magnification. Three trained scientists independently performed blinded scoring. VEGF expression was scored as intensity of the staining reaction in the samples and the immune reactive score (IRS) was calculated as mean IRS score of the three independent observers. VEGF was scored as *negative*, *weak, medium* and *strong* and subsequently categorized into low and high expression grade (Fig. 1a). Absolute numbers of CD31-positive vessels were quantified in four visual microscopic fields per sample and were expressed as mean per sample (Fig. 1b and c). Scores were correlated with N-stage, T-stage, recurrence and 5-year overall survival rate.

### Statistical analysis

Statistical analyses were performed using SPSS© (Statistical Package for the Social Sciences, IBM©) statistical software version 20.0. The level of significance was set to *p* < 0.05. All *P*-values were based on two sided tests. The 5-years-survival curves were plotted according to the Kaplan–Meier method. Significance was tested using the log-rank test. Kruskal-Wallis-test and Mann-Whitney-U test were used to correlate clinical-pathological parameters such as T- and N-stage, respectively, with the number of CD31-positive vessels and VEGF expression. Chi square test was performed to analyze recurrence rates and CD31/VEGF expression in patients with early stage LSCC.

## Results

### Patient cohort

Ninety-seven patients were included in the study and for all patients complete clinical and immunohistochemical data was available. Patients were predominantly male (89%) and tumor stage equally distributed from T1 to T4. VEGF staining was evaluable in 87/97 patients and CD31 data in 89/97 patients. Follow up data were available for 59 (out of 97 patients) in the overall cohort, 34 (out of 50) patients with low T-stage and 44 (out of 67) patients with negative N-stage. Patient demographics are described in Table 1.

### Immunohistochemical scores for VEGF and CD31

VEGF was scored as described above and the number of CD31 positive intratumoral vessels per visual field was quantified and ranged between 0 and 30 (example shown in Fig. 1a+b). Patients were divided into two groups according to the median of CD31-positive vessels, which was calculated as 7.3 (fig. 1c). In subsequent analyses patients with low (< 7) vs high (≥7) vessel count were compared.

### A high number of CD31-positive vessels correlates with low T-stage and negative N-stage

**Fig. 2:**
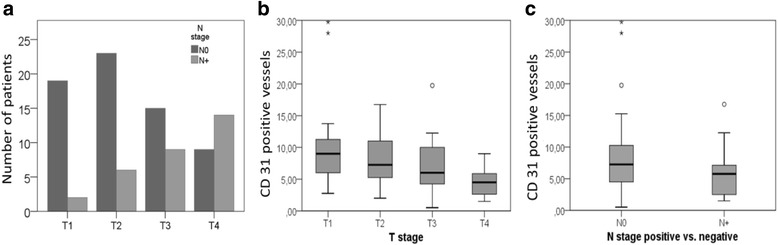
Vessel density is increased in early stage LSCC. T-stage was determined in LSCC patients (*n* = 97). The number of patients without (N0) or with lymph node metastasis (N+) was determined for each T-stage. High T-stage correlated with positive node status (Kruskal-Wallis test; *p* = 0.001) (**a**). The absolute number of vessels per microscopic visual field was determined by immunohistochemistry and correlated with tumor T-stage (**b**) and N-stage (**c**). A high vessel count correlated with low T-stage (Kruskal-Wallis test; *p* = 0.004) and the absence of lymph node metastasis (Mann-Whitney-U test; *p* = 0.028). Data are depicted as box-plots indicating mean and absolute numbers of CD31 positive vessels and error bars

As expected, a positive N-stage significantly correlated with high T-stage in our cohort (*p* = 0.001; Fig. 2a). Interestingly, a high number of CD31-positive vessels was significantly associated with lower T-stage (T1 + T2) (*p* = 0.004; Fig. 2b). In addition, the number of CD31-positive vessels also correlated with N-stage. Interestingly, a high vessel count was rather associated with the absence of lymph node metastasis (N0 status) and not with N+ status (Fig. 2c, *p* = 0.028). These data show that low stage LSCC is characterized by high vessel density. VEGF staining intensity was not significantly associated with T- or N-stage (data not shown).

### Prognostic relevance of angiogenic biomarkers

**Fig. 3:**
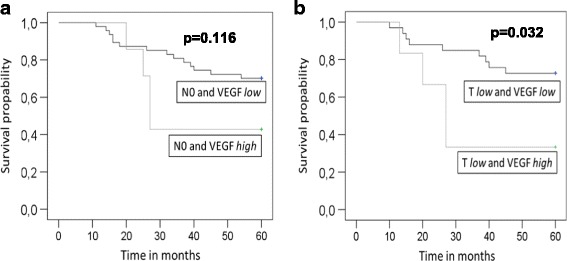
Reduced expression of VEGF correlates with improved survival in early stage LSCC. Patients with negative N-stage (*n* = 67) (**a**) and low T-stage (T1 + T2, *n* = 50) (**b**) were grouped into either VEGF high or VEGF low and further examined in terms of their survival. Kaplan Meier analysis of survival and log rank test were performed

**Fig. 4:**
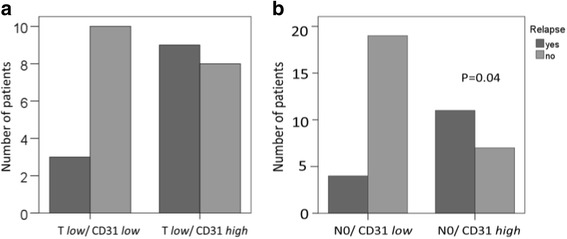
High vessel count is associated with an increased recurrence rate in early stage LSCC. Chi-square-Test was applied to comparerelapse rates and vessel count in patients with (**a**) low T-stage or (**b**) negative N-stage. Patients with a high number of CD31-positive vessels early stage LSCC had a significantly higher relapse rate compared to patients with early LSCC and low number of CD 31-positive vessels. **a** Patients were grouped according to T-stage and vessel count. Number of patients with relapse within 5 years is indicated. Chi-square test; *p* = 0.098. **b** Patients were grouped according to N-stage and vessel count. Number of patients with relapse within 5 years is indicated. Chi-square test; *p* = 0.004

The potential prognostic value of the two biomarkers in the total cohort of LSCC patients was tested. However, neither vessel density nor VEGF expression were associated with overall survival in the total cohort (data not shown). Based on these results a potential correlation of VEGF and CD31 with overall survival was assessed separately in patients with early (T1 and T2, N0) and late (T3 and T4, N+) stage LSCC using Kaplan-Meier analysis. Strong VEGF expression was associated with poor survival in N0 patients, although not reaching statistical significance (*p* = 0.116; Fig. 3a). No association was found in patients with positive N-stage (*p* = 0.927, not shown). Strong VEGF expression also correlated with poor survival in patients with low T-stage (T1 and T2; *p* = 0.032; Fig. 3a). Again no correlation was found in patients with high T-stage (T3 and T4; *p* = 0.846; not shown). Thus, strong VEGF expression was associated with poor survival in early stage but not in late stage LSCC. In contrast, CD31 vessel count was not associated with overall survival in this LSCC cohort, neither in patients with early nor late stage disease (data not shown). Finally, we analyzed a potential link between CD31/VEGF and recurrence-free survival. No correlation was found between the VEGF score and the number of recurrences in early stage LSCC within the 5-year follow-up period. In contrast, our analyses revealed a potential link between vessel count and recurrence rate. As shown in fig. 4a patients with low T-stage and a low number of CD31-positive vessels had a tendency for better recurrence free survival compared to patients with low T-stage and high CD31-positive vessel number, although not reaching statistical significance (Fig. 4a, *p*= 0.098). Low CD31 vessel count significantly correlated with reduced recurrence rate in patients with negative N-stage (*p* = 0.004) (Fig. 4b).

Collectively, these data show that low expression of angiogenic biomarkers seems to be associated with improved clinical outcome in early stage, but not in advanced LSCC.

## Discussion

A potential role for angiogenesis in the prognosis of cancer has been demonstrated in previous studies for different cancer types [5, 25–32]. It was the aim of this study to provide new information about the relation between angiogenesis, tumor stage and prognosis of LSCC.

### Angiogenic biomarkers are particularly associated with early tumor stages

In non-laryngeal HNSCC, studies by Gleich et al. reported an association of angiogenesis with increasing T and N stage in oropharyngeal cancer (OPC) (higher T-stages here defined as T2-T4) [30], but there was no statistical correlation between tumor aggressiveness and tumor angiogenesis in low T stages (here defined as T1) [33]. For LSCC, the literature provides controversial information whether angiogenesis is an early event in LSCC development or whether it is associated with increased tumor stages [24, 29, 34]. Some studies did not show any relation between angiogenesis and prognosis, especially in patients with LSCC [8–11]. In our study we demonstrated a significant correlation of CD31-positive vessels with early T-stages and the absence of LN metastasis (N0 status). There is a controversy in the literature regarding a direct relation between tumor stage and VEGF, another potential marker of angiogenesis. Some studies did not show any association [35] while others revealed significant correlations especially between N-stage and VEGF [27, 36–38]. Interestingly, Wang et al. even reported a strong association of VEGF expression with lymph node metastasis in primary laryngeal carcinomas [27]. In contrast, other studies did not reveal any association of angiogenesis with the presence of LN metastasis [25, 28]. In our analysis there was no direct correlation between T- or N-stage and VEGF.

### Angiogenic biomarkers and their influence on prognosis

As mentioned above the VEGF expression of patients with LSCC is not directly related to T- or N-stage when analyzing the entire cohort. We noted, however, that patients with initial negative N-stage and low T-stage who have a strong VEGF expression showed reduced overall survival. This is in line with findings by Krecicki et al. who reported that a pro-angiogenic tumor microenvironment may promote lymphatic metastasis [6] and consecutively shorter survival. They identified vessel endothelial cells with antibodies against factor VIII and analyzed the vessel density (VD) per image in 55 patients with laryngeal cancer. In their study VD correlated with the existence of nodal metastases (*P* = 0.02) in contrast to histological grading of the tumor or the T-stage. The authors hypothesized that angiogenesis in laryngeal cancer may be of some value in predicting N-stage. In another study, Kupisz et al. found a direct correlation between increased tumor angiogenesis and increased T-stage, histologic grade and a shorter survival rate [25]. Teknos et al. reported that highly-vascularized tumors were associated with worse survival [26]. Williams et al. detected a higher regional recurrence rate in tumors with high angiogenesis and used it as an independent prognostic indicator [39]. Pignataro et al. as well showed that patients with poorly vascularized tumors had a tendency for better prognosis. Similar to our study, no relation between microvessel density and clinical pathological features or prognosis in the later tumor stages was found. The authors suggested that angiogenesis might be an early event in laryngeal tumorigenesis [29]. In our study positive N-stage was not associated with high expression of angiogenic biomarkers in the primary tumor lesion suggesting that that these events are not directly spatiotemporally linked. Instead we found a potential link between angiogenesis and shorter survival exclusively in patients with early stage LSCC. A possible explanation for our findings would be that early stage tumors, which are highly angiogenic prepare the tumor for metastasis. However, these metastasis become clinically detectable at far later time points, when the microenvironment of the primary tumor is no longer characterized by highly expressed angiogenic biomarkers.

In patients with early tumor stage, we also investigated the recurrence rate and found that early recurrence was associated with a higher number of CD31-positive vessels, indicative of a pro-angiogenic microenvironment. Similarly, Bolzoni Villaret et al. analyzed angiogenesis and lymphangiogenesis using immunohistochemistry with antibodies against CD31 and podoplanin. Twenty seven patients with poor outcome were identified and compared with a selected sample of 28 patients. Based on analysis of these groups they concluded that patients with early T-stages of laryngeal cancer showing a pro-angiogenic microenvironment had a significantly higher rate of relapse [2]. Using semiquantitative scoring analysis Murray et al. [40] reported that angiogenesis (CD31 IHC) might be a significant predictor for relapse in patients with negative N-stage. In our study we can substantiate these findings using a large cohort of LSSC patients and exact absolute counting of vessels.

### Relevance of presence of angiogenic biomarkers in early tumor stages in prognosis

In summary in this study we identified VEGF and CD31 as prognostic factors for the clinical outcome of patients with early stage laryngeal cancer. In particular, we demonstrated a significant correlation of CD31-positive vessels with early T-stages and the absence of LN metastasis (N0 status), respectively, but higher relapse rate and we identified strong VEGF expression as a marker of poor survival in patients with a low T-stage and a negative N-stage. Based on these data one could hypothesize that angiogenesis is of special clinical relevance at non-metastatic early tumor stages and determines later tumor progression already at this stage. Based on the data presented in this study we postulate that the quantification of high VEGF and CD31 in early tumor stages could be a useful tool to identify patients with poor prognosis at early tumor and lymph node stages. This could help to improve the clinical management of these patients. As several anti-angiogenic drugs have been developed in the past years [41] these drugs can be considered as additional therapeutic options in high risk LSCC patients with highly angiogenic cancers. Along this line, Beatrice et al. suggested to perform a special anti-angiogenic RCTX in patients with high capillary count even in case of surgical R0 resection. Our results further underscore the hypothesis that a pro-angiogenic microenvironment in early, non-metastatic stages might prepare the tumor for recurrence. Furthermore it indicates that high vessel densities, as well as strong VEGF expression, are characteristic for smaller tumors and might precede the development of metastasis at later stages. These findings would argue in favor of a more radical treatment of a small subgroup of patients with a highly angiogenic tumor microenvironment. However, further studies, ideally in a prospective design, should be performed to substantiate these findings.

## Conclusion

Patients with low T-stage and negative N-stage are generally known to have a good five-year survival rate. However, our study identified a subgroup of patients with strong VEGF expression and high CD31-positive vessel number, which was linked to poor prognosis and increased risk for relapse. Thus, our study provides further evidence that quantification of angiogenesis in a subgroup of patients with laryngeal cancer could help to predict patient outcome and to better guide therapeutic decisions in this cancer entity.

## Abbreviations

IRS: Immune Reactive Score
LN: Lymph Node
LSCC: laryngeal squamous cell carcinoma
VEGF: vascular endothelial growth factor
